# The accuracy and characteristics of gastric cancer treatment information in the national data of the hospital-based cancer registry

**DOI:** 10.1093/jjco/hyae014

**Published:** 2024-02-09

**Authors:** Manami Fujishita, Naoki Sakakibara, Takahiro Higashi, Tomone Watanabe, Hiraku Kumamaru, Hiroaki Miyata

**Affiliations:** Division of Health Services Research, National Cancer Center, 5-1-1, Tsukiji, Chuo-ku, Tokyo 104-0045, Japan; Department of Health Policy and Management, Graduate School of Medicine, Keio University, 35, Shinanomachi, Shinjuku-ku, Tokyo 160-8582, Japan; Department of Public Health and Health Policy, Graduate School of Medicine, The University of Tokyo, 7-3-1, Hongo, Bunkyo-ku, Tokyo 113-0033, Japan; Division of Health Services Research, National Cancer Center, 5-1-1, Tsukiji, Chuo-ku, Tokyo 104-0045, Japan; Department of Public Health and Health Policy, Graduate School of Medicine, The University of Tokyo, 7-3-1, Hongo, Bunkyo-ku, Tokyo 113-0033, Japan; Division of Health Services Research, National Cancer Center, 5-1-1, Tsukiji, Chuo-ku, Tokyo 104-0045, Japan; Department of Public Health and Health Policy, Graduate School of Medicine, The University of Tokyo, 7-3-1, Hongo, Bunkyo-ku, Tokyo 113-0033, Japan; Division of Health Services Research, National Cancer Center, 5-1-1, Tsukiji, Chuo-ku, Tokyo 104-0045, Japan; Department of Public Health and Health Policy, Graduate School of Medicine, The University of Tokyo, 7-3-1, Hongo, Bunkyo-ku, Tokyo 113-0033, Japan; Department of Healthcare Quality Assessment, Graduate School of Medicine, The University of Tokyo, 7-3-1, Hongo, Bunkyo-ku, Tokyo 113-8655, Japan; Department of Health Policy and Management, Graduate School of Medicine, Keio University, 35, Shinanomachi, Shinjuku-ku, Tokyo 160-8582, Japan; Department of Healthcare Quality Assessment, Graduate School of Medicine, The University of Tokyo, 7-3-1, Hongo, Bunkyo-ku, Tokyo 113-8655, Japan

**Keywords:** cancer registry, gastric cancer, diagnosis-procedures combination, GI-stomach-basic

## Abstract

**Objective:**

The hospital-based cancer registry is used extensively for research to support cancer control activities by providing an overview of how cancer treatments are provided nationwide. This study aimed to shed light on the quality and characteristics of treatment data in the hospital-based cancer registry using the linked dataset on gastric cancer.

**Methods:**

Using the nationally linked data of the hospital-based cancer registry and the health services utilization data, the treatment data in the hospital-based cancer registry for patients who were newly diagnosed with gastric cancer in 2016 and 2017 and received the first course of treatment at their own institutions were examined. The agreement rates between registry data and utilization data were analyzed by stage, treatment, age, period from the date of diagnosis to the date of treatment and hospital type.

**Results:**

The sensitivity of open surgery, laparoscopic surgery and endoscopic treatment tended to decrease in advanced stages, whereas the sensitivity of chemotherapy and radiation therapy increased. Specificity was high for all treatments and stages, at ˃90%. Sensitivity by age was slightly different for chemotherapy and radiation therapy, but specificities did not differ.

For all treatments, the longer the time from diagnosis to treatment implementation, the higher the coverage rate.

**Conclusions:**

The hospital-based cancer registry recorded the treatment performed appropriately. It is necessary to interpret the data from the hospital-based cancer registry whilst keeping in mind that, chemotherapy and radiation therapy are registered less frequently than surgical treatments administered.

## Teaser

Hospital-based cancer registry appropriately recorded the treatment according to the registration rules. The rules of registration period also appeared practical.

## Introduction

Cancer registries are databases that collect basic information such as the type of cancer, its progression, treatment and prognosis in a population defined by the medical facilities or geographic locations. They are used worldwide to support cancer control and to improve the quality of cancer care. In Japan, there are two types of cancer databases: a population-based cancer registry (PBCR) and a hospital-based cancer registry (HBCR). The latter provides a registry of cancer patients at each hospital and describes the actual state of medical care based on the Union for International Cancer Control stages and treatment information, which are not available in the PBCR, in order to improve of the quality of cancer care.

Japan has been designating cancer care hospitals that satisfy certain standards since 2006. The standards include the provision of specialized cancer treatment, establishment of a local relationship with other facilities for cancer treatment, and operation of patient consultation centers, as well as the maintenance of an HBCR and annual submission of data to the National Cancer Center. As of 1 April 2023, there were 456 Designated Cancer Care Hospitals and 47 hospitals designated as Cancer Liaison hospitals (1). Furthermore, HBCRs have been operated at certain specialized facilities other than those designated by the Ministry of Health, Labour and Welfare and an analysis using the 2018 national data of HBCRs reported that 70.9% of newly diagnosed cancer patients in Japan (all sites; excluding C00-C96 epithelial cancer) were covered by HBCRs (2).

HBCRs provide useful information about cancer care at both the facility level and the national level. They record basic information on cancer such as location, histology and stages, as well as the first course of treatment provided at the facility. The first-course treatment is defined as ‘treatment given to reduce or dissect cancerous tissue of the primary tumor or metastases, following the initial diagnosis of the tumor.’ Because these data are submitted to the National Cancer Center, at about the middle of the following year (June–August), treatment after the submission date may be truncated, especially for the cases with diagnosis near the year-end. Therefore, to ensure homogeneity of data, only treatments initiated within 5 months of diagnosis at the hospital are to be included in the statistical reports, regardless of the time of diagnosis during the year. However, the validity of this rule has not recently been examined. This should be verified in light of the recent increased use of multidisciplinary treatment combining multiple therapies. If some initial treatment is initiated ˃6 months after diagnosis, the data are not counted, but if a large percentage of patients are so treated, a change in the rule may be needed.

Detailed data on treatment are collected in a separate system, called the ‘Diagnosis-procedures combination (DPC) survey.’ It aims to evaluate the impact of the introduction of the DPC system, a system that provides prospectively determined reimbursement per day, and is equivalent to the fee-for-service-based health insurance claims data, including the performance of laboratory tests, imaging studies, procedures and drugs prescribed. These data are collected secondarily and linked to the HBCR to measure the quality of cancer care by adherence to the standards of care (Quality Measurement Project). Participation in the Quality Measurement Project is voluntary, but ~80% of cancer care hospitals (77% in 2016 and 83% in 2017) submit data (3). These data have been used in the past for various data quality assessments in conjunction with the HBCR; Takaoka et al. reported a tumour-node-metastasis (TNM) classification concordance rate of 77% (95% confidence interval: 75–79%) for five major cancers in four institutions (4). Further, Okuyama et al. extended the analysis to the HBCR data provided by 231 hospitals and DPC data linked to it, found that the TNM classification of patients aged 20 years or older diagnosed with stomach, colorectal, liver, lung and breast cancer in 2013 had an agreement rate of 80.6% (5). The same types of datasets can be used to evaluate the treatment information record in the HBCR.

The present study examined the extent to which data from the HBCR reflects actual medical treatment, as well as the appropriateness of the 5-month inclusion rule using the linked dataset. This analysis focused on gastric cancer to interpret the findings in a clinical context.

## Methods

### Data source

National data from the 2016 and 2017 HBCR (diagnosis period from January 2016 to December 2017) and DPC health service utilization survey data (inpatient and outpatient practice data from October 2015 to March 2018), from 476 facilities in 2016 and 532 facilities in 2017 that provided linkable data were used. Data from 498 009 patients were included. Of these, data from a total of 570 facilities were analyzed for 135 046 patients who were newly diagnosed with gastric cancer between 1 January 2016, and 31 December 2017, and who received initial treatment at their own facility. Cases involving patients that had not been treated at the institution, those with an unknown stage, and those with unknown dates of diagnosis and treatment were excluded from the analysis.

### Data analysis

First, how well the national data of the HBCR accurately extracted the treatments that were performed was evaluated using the DPC data linked to the national data of the HBCR as the gold standard for the cases diagnosed with gastric cancer that received their first treatment at their institutions between 2016 and 2017. The accuracy of the registry was defined as the percentage of concordance with DPC data. The concordance rates (sensitivity and specificity) with the HBCR data were analyzed by overall stage, treatment (open surgery, laparoscopic surgery, endoscopic treatment, chemotherapy and radiation therapy), age, period from the date of diagnosis to the date of treatment and hospital type. The overall stage summarized the clinical and pathological stages by pathological stages when available, supplemented by clinical stages when pathological stages were not available. DPC data were selected as the gold standard because they have a wider data collection scope than the HBCR, and because specific medical treatment details are automatically output from electronic medical records and other sources in accordance with medical fee information, thus minimizing the risk of omission.

For each treatment for gastric cancer, the appropriate range of treatment was defined in the DPC data, sensitivity was defined as the ratio of treatments ‘performed’ in the DPC data that were also ‘performed’ in the national HBCR data, and specificity was defined as the ratio of treatments ‘not performed’ in the DPC data that were also ‘not performed’ in the HBCR data. According to the rules used in the HBCR annual reports, the HBCR data were defined as medical treatment performed within 150 days from the date of diagnosis, and data that did not conform to this definition were corrected. On the other hand, specificity was defined as the proportion of those for which absence of respective treatment in the DPC data was correctly recorded in the HBCR data. Because first-course treatment cannot be distinguished from treatment added in response to the clinical condition in the DPC data, simply the presence/absence of treatment in the DPC data was examined and the distinction was taken into account in the discussion of the findings.

To examine the appropriateness of 150 days as the cut-off for the inclusion of the treatment data, the distribution of the days from the date of diagnosis to the first performance of the respective treatments was examined. The proportion of respective treatments provided within 120 150 and 180 days was first calculated, and the graph of case distribution was drawn. Since the trend of the distribution was unstable, the 10-day moving average of cases per day was calculated.

All statistical analyses were performed using Stata 16.0 (Stata Corporation, College Station, TX, USA).

### Ethical considerations

This study was approved by the Institutional Review Board of the National Cancer Center, Japan (Approval number: 2020–528).

## Results

### Sample characteristics

Sample characteristics are shown in Table 1. In total, 95 526 cases (70.7%) involved male patients, and the median age was ~72 years for both sexes. With respect to stage, Stage I was the most common (85 933 cases, 63.6%), followed by Stage IV (21 859 cases, 16.2%). In terms of treatment, endoscopic treatment was the most common, at 54237 (40.2%), followed by chemotherapy at 29 069 (21.5%), open surgery at 28 901 (21.4%), laparoscopic surgery at 26 456 (19.6%) and radiation therapy at only 0.3% (469 cases). Regarding type of hospital, 380 (66.7%) were designated cancer care Hospitals, and 190 (33.3%) were others, with designated cancer care hospitals accounting for ~80% of the total number of cases, at 108 978 (Table 2).

**Table 1:** Sample characteristics

Patients’ Characteristics	n	%
Number of patients	135 046	100
Male	95 526	70.7
Female	39 520	29.3
Age	Male 72.0 (9.7) 0–103	
Mean (SD) Range	Female 72.3 (11.8) 18–108	
Stage		
I	85 933	63.6
II	12 073	8.9
III	13 350	9.9
IV	21 859	16.2
Unkown	1831	1.4
Cancer treatment^a^		
Open Surgery	28 901	21.4
Laparoscopic surgery	26 456	19.6
Endoscopic treatment	54 237	40.2
Chemotherapy	29 069	21.5
Radiation therapy	469	0.3

^a^Including duplicates.

**Table 2:** Sample characteristics in 570 hospitals

Type of hospital	N of patients	N of hospitals	%
Designated cancer care hospitals	108 978	380	80.7
Others	26 068	190	19.3
Total	135 046	570	100.0

Data were corrected by defining treatment performed within 150 days of diagnosis as ‘performed’ and treatment performed later as ‘not performed,’ even if it was performed. After adjusting for HBCR data, the results were as follows: open surgery 1.59% (468/26 930), laparoscopic surgery 1.76% (473/26 929), endoscopic treatment 1.21% (667/54 904), chemotherapy 1.11% (327/29 396) and radiation therapy 4.09% (20/489).

Sensitivity by stage and treatment is shown in (Table 3). Overall, ˃95% of the surgical and endoscopic procedures present in the DPC data were captured in HBCR treatment data, whereas 87.8% of chemotherapy was recorded in the HBCR. Only a few patients (n = 432) received radiation therapy, and a further minority was recorded in the HBCR. The sensitivity of open surgery was high, at greater than 97% for all stages. The sensitivity of laparoscopic surgery was the highest in Stage II, at 96.4% (n = 3434), and the sensitivity tended to decrease as the stage progressed. The sensitivity of endoscopic treatment was the highest in Stage I, at 98.8% (n = 52 470), and it decreased as the disease progressed, whereas the sensitivity of chemotherapy increased as the disease progressed and was the highest in Stage IV, at 97.9% (n = 13 965).

**Table 3:** Sensitivity by stage cancer treatment

	Sensitivity (%)				
	Open surgery	Laparoscopic surgery	Endoscopic treatment	Chemotherapy	Radiation therapy
Total	97.8	95.4	98.7	87.8	19.2
	(26 757/27 359)	(24 928/26 137)	(52 672/53 392)	(27 900/31 770)	(432/2253)
Stage I	96.7	95.3	98.8	25.6	4.6
	(8067/8341)	(19 028/19 956)	(52 470/53 094)	(834/3256)	(50/1034)
Stage II	98.5	96.4	72.4	90.6	24.3
	(6548/6645)	(3434/3561)	(110/152)	(4950/5465)	(37/152)
Stage III	98.6	95	59.3	94	30.3
	(8598/8724)	(2050/2158)	(32/54)	(8216/8737)	(43/142)
Stage IV	97.2	90.1	40.5	97.9	36.7
	(3516/3618)	(411/456)	(17/42)	(13 965/13 989)	(295/804)

Specificity by stage and treatment is shown in Table 4. Specificity was high for all treatments and stages, at 90% or higher, but it was especially high for radiotherapy, at almost 100%.

**Table 4:** Specificity by stage cancer treatment

	Specificity (%)				
	Open surgery	Laparoscopic surgery	Endoscopic treatment	>Chemotherapy	Radiation therapy
Total	98	98.6	98.1	98.9	100
	(105 540/107 684)	(107 378/108 906)	(80 086/81 651)	(102 099/103 268)	(132 749/132 786)
Stage I	98.7	98.6	95.4	99.9	100
	(76 579/77 590)	(65 064/65 975)	(31 332/32 839)	(82 565/82 672)	(84 845/84 847)
Stage II	94.8	97.4	99.9	98.4	100
	(5145/5428)	(8290/8512)	(11 903/11 920)	(6502/6606)	(11 914/11 919)
Stage III	92.3	98.2	99.9	94.5	99.9
	(4267/4625)	(10 995/11 192)	(13 278/13 294	(4357/4613)	(13 201/13 208)
Stage IV	97.3	99.1	99.9	91.3	99.9
	(17 755/18 241)	(21 206/21 402)	(21 802/21 817	(7186/7869)	(21 029/21 052)

The sensitivity and specificity by treatment when stratified by age into groups with patients age 75 years or older and those age ˂75 years are shown in Table 5. The sensitivity of open surgery, laparoscopic surgery and endoscopic treatment did not differ between the two groups, whereas the sensitivity of chemotherapy was 69.8% (n = 7994) for those aged 75 years or older, compared with 82.1% (n = 20 586) for those younger than age 75 years. On the other hand, the sensitivity of radiation therapy was 11.0% (n = 194) in those over 75 years of age or older, compared with 9.1% (n = 241) in those under age 75 years. Specificity did not differ between the two groups.

**Table 5:** Sensitivity and specificity by age at cancer treatment

	Sensitivity (%)				
Age (y)	Open surgery	Laparoscopic surgery	Endoscopic treatment	Chemotherapy	Radiation therapy
≧75	95.5	91.5	96.2	69.8	11
	(11 427/11 968)	(7868/8600)	(24 275/25 235)	(7994/11 454)	(194/1757)
<75	93.3	92.2	96.8	82.1	9.1
	(15 351/16 445)	(17 072/18 512)	(29 291/30 259)	(20 586/25 081)	(241/2659)
	Specificity (%)				
Age (y)	Open surgery	Laparoscopic surgery	Endoscopic treatment	Chemotherapy	Radiation therapy
≧75	98.5	99.1	99.2	99.7	100
	(46 540/47 271)	(50 193/50 640)	(33 728/34 002)	(47 658/47 781)	(57 471/57 481)
<75	97.7	98.1	99.1	99.3	100
	(57 967/59 359)	(56 222/57 291)	(45 150/45 547)	(50 356/50 722)	(73 118/73 142)

The concordance rates between the designated cancer care hospitals and others for each treatment did not differ significantly between the hospitals, except for radiation therapy (Table 6). Sensitivity in radiation therapy was 9.3% (n = 359) in designated cancer care hospitals, compared with 13.6% (n = 76) in others.

**Table 6:** Sensitivity and specificity by hospital type

	Sensitivity		Specificity	
	(%)	n	(%)	n
**Open surgery**				
Designated cancer care hospitals	94.2	21 172/22 473	98	84 783/86 503
Others	94.4	5606/5940	98	19 724/20 127
**Laparoscopic surgery**				
Designated cancer care hospitals	92	20 629/22 422	98.5	85 261/86 553
Others	91.9	4311/4690	99	21 154/21 378
**Endoscopic treatment**				
Designated cancer care hospitals	96.4	43 808/45 433	99.2	63 013/63 542
Others	97	9758/10 061	99.1	15 865/16 007
**Chemotherapy**				
Designated cancer care hospitals	78.3	23 327/29 799	99.4	78 733/79 173
Others	78	5253/6736	99.7	19 281/19 330
**Radiation therapy**				
Designated cancer care hospitals	9.3	359/3856	100	105 092/105 119
Others	13.6	76/560	100	25 497/25 504

**Figure 1:**
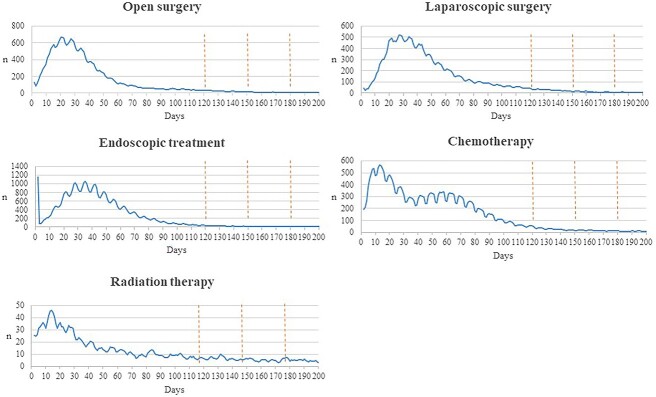
Distribution of patients started with treatment by days after diagnosis (Simple 10-day moving average).

The moving average of the number of cases by treatment from the date of diagnosis to the date of treatment in the DPC data is shown in Fig. 1. The percentages of the cases performed up to 120, 150 and 180 days are shown in Table 7. For all treatments, the longer the time from diagnosis to treatment implementation, the higher the coverage rate. In particular, for laparotomy, laparoscopic surgery and endoscopic therapy, coverage was ˃96% at 150 days after diagnosis. For chemotherapy, the coverage rate exceeded 90% at 150 days after diagnosis, whereas for radiation therapy, the coverage rate was as low as 60.4%, even 180 days after diagnosis.

**Table 7:** Coverage of treatment performed up to the 120, 150 and 180 days

	Sensitivity		Specificity	
	(%)	n	(%)	n
**Open surgery**				
Designated cancer care hospitals	94.2	21 172/22 473	98	84 783/86 503
Others	94.4	5606/5940	98	19 724/20 127
**Laparoscopic surgery**				
Designated cancer care hospitals	92	20 629/22 422	98.5	85 261/86 553
Others	91.9	4311/4690	99	21 154/21 378
**Endoscopic treatment**				
Designated cancer care hospitals	96.4	43 808/45 433	99.2	63 013/63 542
Others	97	9758/10 061	99.1	15 865/16 007
**Chemotherapy**				
Designated cancer care hospitals	78.3	23 327/29 799	99.4	78 733/79 173
Others	78	5253/6736	99.7	19 281/19 330
**Radiation therapy**				
Designated cancer care hospitals	9.3	359/3856	100	105 092/105 119
Others	13.6	76/560	100	25 497/25 504

## Discussion

Using the linked dataset of the HBCR and DPC-survey health services utilization data for gastric cancer, we characterized the information on the first-course treatments on the HBCR recorded according to the operational rules of collection. The sensitivity and specificity of the information were found to be generally high for surgical procedures and endoscopy, but the sensitivities for chemotherapy and radiation therapy were relatively low. Since the treatment information in the DPC survey data do not distinguish the first-course treatment from the treatment added during the clinical course, the low sensitivity may simply mean that much of the chemotherapy and radiation therapy were not planned at the start of therapy right after diagnosis (and thus not considered first-course treatment). In any case, the number of patients who received radiation therapy was small, because radiation therapy is rarely indicated for treatment of gastric cancer.

In the interpretation of the results, it is important to bear in mind the difference in the recording rules between the HBCR and the DPC survey data. Whereas the HBCR records only first-course treatments, which are planned at the time of diagnosis and implemented at the registering institution, the DPC survey data include all the treatment provided in the recording hospitals. The distribution of days on which respective treatments were started and the coverage were examined by the cut-off days. The high coverage at day 150, the presence of little change at day 180 and the almost flat curve in the graphs indicates that the current cut-off of day 150 is appropriate. Although extending the registration period in the HBCR to day longer than 150 would slightly increase the accuracy of registration for chemotherapy and radiation therapy, the trend of coverage rates suggest that the benefits would not outweigh the delay in the reporting of the HBCR.

According to the treatment guidelines for gastric cancer in Japan (6), for stage I gastric cancer without lymph node metastasis, whether endoscopic treatment is indicated is first considered, and if not, open or laparoscopic surgery is recommended as standard treatment. For Stage I or higher gastric cancer with lymph node metastasis, the guidelines recommend open or laparoscopic surgery or open surgery after preoperative chemotherapy or adjuvant chemotherapy following open surgery. Furthermore, for Stage IV gastric cancer with distant metastasis, chemotherapy is generally recommended, but radiation therapy or palliative surgery may also be considered.

According to the ESMO clinical practice guidelines (7), endoscopic resection is recommended for very early stage gastric cancer (T1a) under certain conditions. T1 tumours that do not meet the criteria for endoscopic resection require surgery; perioperative therapy and radical gastrectomy are recommended for Stage IB-III gastric cancer, although radical gastrectomy is indicated in Stage IB-III cases. Perioperative (preoperative and postoperative) chemotherapy is also recommended for patients with resectable gastric cancer of Stage IB or higher. Gastrectomy is not recommended for metastatic gastric cancer unless necessary for palliation of symptoms.

Based on the above findings, the results of the present analysis show that the sensitivity of surgery as a possible treatment choice was high for all stages of disease. In addition, the sensitivity of endoscopic treatment decreased for Stage I or higher, and the sensitivity of chemotherapy and radiation therapy increased as the stage of the disease progressed, indicating that the treatments that were ‘performed’ in the DPC data and also registered in the HBCR showed the same trend as standard treatment. Furthermore, the specificity was high for all stages and treatments, suggesting that treatments that were considered ‘not performed’ in the DPC data were appropriately registered as ‘not performed’ in the HBCR as well.

The unique findings about radiation therapy may merit mention. The sensitivity of HBCR for radiation therapy is generally low, and the analysis by hospital type showed that the sensitivity of radiotherapy was lower at the designated cancer care hospitals. The reasons for these findings are not apparent, but they probably arise from the fact that radiation therapy is seldom part of standard treatment for gastric cancer. The HBCR does not record them when they are not a first-course treatment (i.e. unplanned at the diagnosis), and designated cancer care hospitals distinguish first-course treatment versus unplanned but added treatment more rigorously and drop the latter treatment appropriately. This may need further investigation to enhance the accuracy of the registry information, with inclusion of other cancers in the analysis.

At present, HBCR data and DPC data are anonymized and collected separately, so linking these data is not easy and will require reprocessing and resubmission with the cooperation of many hospitals. However, the results of the present study showed that the HBCR data were valid, and it was thought that, if only treatment were to be looked at, the data could accurately describe the presence or absence of treatment performed without linkage to DPC data. If a data collection system is established, and linkage can be easily implemented in the future, there may be less need to collect treatment information in the HBCR. In this way, the timing of data collection could be made faster.

The present study has several limitations. First, the DPC data simply recorded billing codes and do not capture clinical context. For example, it is possible that treatments were performed for other co-existent cancers in the DPC data. Such treatments were not recorded in the in-hospital cancer registry, resulting in the discrepancy. We need to be aware that the concordance will never be 100% because of these factors. Second, since the HBCR and DPC data do not contain the treatments that were provided in institutions other than the registering hospital, the data differ from population-based data such as United States Surveillance Epidemiology and End Result (SEER) registry that includes ‘All treatments administered to the patient after the original diagnosis of cancer in an attempt to destroy or modify the cancer tissue’ (8). Finally, radiation therapy, which is rarely implemented as a standard treatment for gastric cancer, was difficult to evaluate due to the small number of cases, which was considered a limitation due to the treatment characteristics of gastric cancer. Similar validation should be conducted for other cancer types, such as breast and lung cancer, in order to evaluate chemotherapy and radiation therapy.

## Conclusions

Despite the limitations, this study generally confirmed that the HBCR adequately reflects the treatments performed based on the definition of first-course treatment. Although the current submission timing for the HBCR is considered appropriate, it is necessary to interpret the data from the HBCR whilst keeping in mind that chemotherapy and radiation therapy are registered in smaller numbers than the actual treatments administered.

## Funding

This work was supported by the Cancer Research and Development Fund of National Cancer Centre, Japan.

## Conflict of interest statement

All authors have no conflicts of interest to disclose.
